# Effect of Kinesiotape Applications on Ball Velocity and Accuracy in Amateur Soccer and Handball

**DOI:** 10.1515/hukin-2015-0114

**Published:** 2015-12-30

**Authors:** Carsten Müller, Mirko Brandes

**Affiliations:** 1 Institute of Sport Science, Work Unit Human Performance and Training in Sports, University of Münster, Germany; 2 Institute of Sport Science, Carl von Ossietzky University of Oldenburg, Germany

**Keywords:** athletic performance, proprioception, elastic bandage, sports equipment, soccer

## Abstract

Evidence supporting performance enhancing effects of kinesiotape in sports is missing. The aims of this study were to evaluate effects of kinesiotape applications with regard to shooting and throwing performance in 26 amateur soccer and 32 handball players, and to further investigate if these effects were influenced by the players’ level of performance. Ball speed as the primary outcome and accuracy of soccer kicks and handball throws were analyzed with and without kinesiotape by means of radar units and video recordings. The application of kinesiotapes significantly increased ball speed in soccer by 1.4 km/h (p=0.047) and accuracy with a lesser distance from the target by −6.9 cm (p=0.039). Ball velocity in handball throws also significantly increased by 1.2 km/h (p=0.013), while accuracy was deteriorated with a greater distance from the target by 3.4 cm (p=0.005). Larger effects with respect to ball speed were found in players with a lower performance level in kicking (1.7 km/h, p=0.028) and throwing (1.8 km/h, p=0.001) compared with higher level soccer and handball players (1.2 km/h, p=0.346 and 0.5 km/h, p=0.511, respectively). In conclusion, the applications of kinesiotape used in this study might have beneficial effects on performance in amateur soccer, but the gain in ball speed in handball is counteracted by a significant deterioration of accuracy. Subgroup analyses indicate that kinesiotape may yield larger effects on ball velocity in athletes with lower kicking and throwing skills.

## Introduction

In recent years a novel concept of kinesiotape (KT), also known as dynamic or medical taping, has gained interest in sports and many areas of general medicine as well as physiotherapy. For various competitive and recreational sports such as soccer, handball, volleyball or baseball, KT represents an additional part of prevention, rehabilitation, exercise therapy and performance optimization (Hsu et al., 2009; Briem et al., 2011; Merino-Marban et al., 2013). Similarly, treatment and aftercare approaches have been introduced in orthopedics, trauma surgery and oncology by means of KT (Yasukawa et al., 2006; Thelen et al., 2008; Tsai et al., 2009).

In sports like soccer and handball, muscle strength is a key component that determines physical performance of an athlete, with shooting and throwing velocity and accuracy being most important for success. For example, maximizing the hip flexor and knee extensor moment seems to be an important factor for increasing foot velocity on impact and thus, allows to attain maximum ball speed in soccer (Lyle et al., 2011; Young and Rath, 2011). Similarly, the optimal proximal to distal transfer of momentum with the braking action of the drive leg to provide a stable base of support, as well as high levels of upper extremity muscle strength and power are prerequisites for success in handball (Raeder et al., 2015; Wagner et al., 2012b; Rousanoglou et al., 2014). In this context, one of the traditionally assumed qualities of KT applications in sports is to impact muscle tone, with the direction of the tape arrangement being decisive: taping from the anatomical origin to the insertion is supposed to increase muscle tone, while the reversed attachment is supposed to decrease muscle tone (Vered et al., 2015). However, evidence for both effects is not clear, since recent studies failed to demonstrate the purported inhibitory effect (Vered et al., 2015; Gusella et al., 2014; Vercelli et al., 2012).

Evidence for facilitating effects of KT applications both in the lower and upper extremity in healthy subjects remains controversial, as recent studies failed to demonstrate any changes in peak torque and surface electromyography (sEMG) activity of the quadriceps muscle in women (Lins et al., 2013), non-injured young athletes (Fu et al., 2008) or subjects participating in nonprofessional sports activities (Vercelli et al., 2012). Similarly, KT applied to the forearm did not alter maximal grip strength (Chang et al., 2010). In summary, a recent meta-analysis suggested that the application of KT with regard to muscle contraction facilitation has no or negligible effects in healthy subjects irrespective of muscle-groups taped (Csapo and Alegre, 2015).

In contrast, some studies reported a significant increase in peak torque by KT both in the lower (Slupik et al., 2007) and upper extremity (Hsu et al., 2009). Although KT applied to the vastus medialis did not alter muscle peak torque evaluated by means of an isokinetic dynamometer, a significantly shorter time to peak extension torque was found, being relevant for kicking and throwing ball velocity in team sports (Wong et al., 2012). Finally, a sEMG study demonstrated an increase in isometric strength and gastrocnemius muscle activity by means of KT applications (Csapo et al., 2012). Of note, the degree of performance-enhancement was dependent on baseline strength with weaker participants demonstrating greater benefits from the taping intervention.

In addition to a potential increase in muscle tone, only few studies evaluated the potential impact of KT in sports specific performance. Though facilitation effects could be demonstrated in the calf muscles, these did not result in an increased height in vertical jumps (Huang et al., 2011) or performance in the single-leg hop test (Firth et al., 2010). Moreover, KT applied to the gastrocnemius, hamstring, rectus femoris, and iliopsoas muscles failed to improve jumping performance between taped and untaped conditions in female track and field athletes (Schiffer et al., 2015). Nakajima and Baldridge (2013) utilizing KT as a tendon correction technique applied to the ankle and surrounding muscles, demonstrated that postural control improved in female non-injured athletes, a substantial skill in team sports like soccer and handball when transferring energy from proximal to distal segments during kicking and throwing movements. Thus, Drouin et al. (2013) acknowledged minor positive effects of KT, but highlighted that evidence was still lacking to generally support the use of KT. Given the controversial findings, the aim of the present study was to examine the effectiveness of KT on performance in amateur soccer and handball players. Consequently, ball speed was judged the primary outcome and accuracy the secondary outcome. Furthermore, the players’ level of performance was taken into account.

## Material and Methods

### Participants

**Figure 1:**
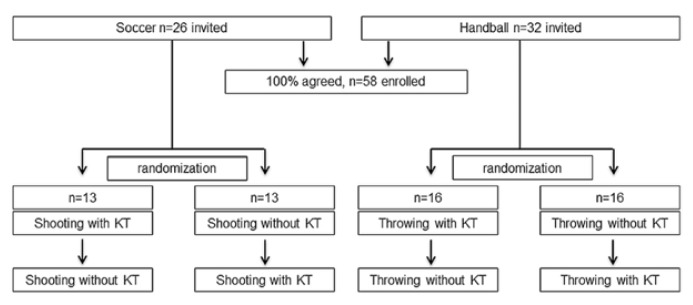
Study protocol

A total of 58 skilled male athletes were asked to participate and all of them were enrolled in this study, including 26 active soccer players aged 23.9 ±5.6 years with 15.5 ±7.1 years of playing experience, and 32 handball players at the age of 27.3 ±8.5 years with an average training experience of 13.8 ±1.2 years (Figure 1).

Soccer players were competing in the mid-level of German amateur leagues. They participated in approximately 2–3 training sessions per week and 26–30 match days during the season. Seventeen soccer players had not previously had any experience with tape applications. Two players had experience with kinesiotape, six participants were experienced with white athletic tapes, and one player was experienced with both types of tapes.

Handball players were competing in the mid-level of German amateur leagues. They practiced approximately 1–3 days per week and participated in approximately 26 match days during the season. Eighteen handball players already had prior experience with tape applications. Ten of them were experienced with kinesiotape, four of them with white athletic and four were experienced with both types of tape.

All procedures of the study were in accordance with the ethical principles for medical research involving human subjects as published by the World Medical Association in the Declaration of Helsinki. Informed written consent was obtained from each person prior to the participation in this study. Exclusion criteria were acute injuries, trauma within the previous three months and any current painful conditions. During the test all participants wore their normal sportswear. Soccer players wore indoor shoes, as all tests were carried out indoor to prevent irregularities from the ground and wind.

### Procedures

Examinations were held at the usual times of the team training between 7 and 10 pm. During the test procedure constant room temperature was guaranteed. Furthermore, participants were advised not to undergo any further tests or strenuous workouts on the day before and the day of the examination.

All participants were randomly assigned to one of two groups. Randomization was performed by means of picking lottery numbers immediately before the start of the examination. This procedure prevented that the test subjects could prepare themselves for the upcoming performance with or without KT application. The first group performed the tests after application of KT and repeated them after a recreational phase of at least 60 min without KT. The second group passed the trials in reverse order.

**Figure 2 a, b:**
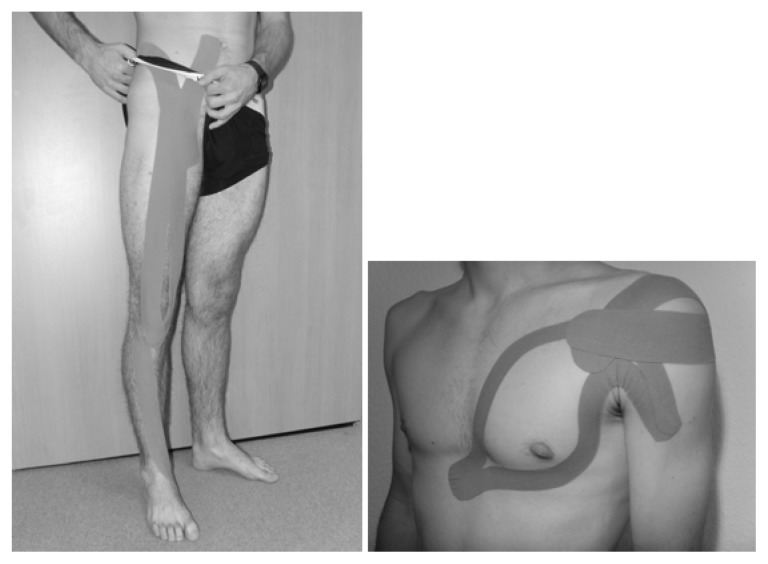
KT applications in soccer and handball

In the current trials the Towatek^®^ kinesiotape (Towatek Korea Co., LTD., Gyomun-dong, South Korea) was used. All tape arrangements were put on the hairless or shaven skin by one KT-experienced physiotherapist and sports scientist. In order to achieve an increase in the muscle tone, KT was applied with a stretch of 20–30% of its original length from the anatomical origin to the insertion of the muscle. The muscles m. tibialis anterior, m. quadriceps femoris and m. iliopsoas, being active during the whole movement of shooting (Dorge et al., 1999; Young and Rath, 2011), were supposed to have the greatest importance for kicking in soccer (Figure 2a). For throwing in handball the m. pectoralis major and m. subscapularis were judged as essential and hence, considered for the KT arrangement. Since the subscapularis cannot be taped directly due to its anatomical location, an application to support internal rotation was applied according to Figure 2b, which was hereafter referred to as KT subscapularis. The base was attached caudal to the coracoid process. The athlete then externally rotated the shoulder, before the tape ran out on the back of the scapula. Besides, the comfort of the KT arrangements on the skin was assessed by means of a questionnaire in order to examine a possible effect of positive or negative perceptions on shooting and throwing performance.

All subjects carried out their individual warm-up routines of approximately ten minutes previously to both rounds with the aim to be well prepared for the task ahead. These routines were identical in both rounds to avoid side effects of different warm-up routines on the outcome. For trials evaluating KT applications on performance, the tape was applied prior to the warm up routine. In measurements without KT, the warm up was also performed without KT.

### *Soccer* goal shooting

The kicking task is in accordance with a goal shot during the game, with two parameters being of great importance for success, namely achieving high ball velocity and accuracy (Andersen and Dorge, 2011). The experimental setup consisted of goal shots from a distance of 8 m and a run-up of 2 m, using a straight approach. A squared grid with black stripes and a filled red square in the middle was fixed on a large soft mat (3×2 m; length × height), placed in a handball goal, to define the target. The size of each square was 30×30 cm. Seven goal shots were evaluated with the objective to strike the red square of the target mat with maximum velocity. Players were instructed to shoot at the highest speed possible. If players missed the mat, the shot was excluded.

### Handball goal throwing

For the analysis of goal throwing performance, a distance of 7 m with a run-up of 2–3 steps was chosen, as this procedure is characteristic for throwing in handball. All players were instructed to throw with maximum velocity and that the premise was on speed, not accuracy. For accuracy analysis, a Perspex (break-proof plexiglass), measuring 1 × 1 m, was used as a target and fastened in the right upper corner of the goal. A crosshair in the center of this target was surrounded by four circles within a distance of 10 cm. Again, 7 throws were analyzed.

### Measures

Ball velocity (km/h) in soccer and handball was determined by means of a radar device (SpeedTrac XTM, EMG Companies Inc., Prescott, USA). For accuracy analysis in soccer and handball, shots and throws were recorded with the help of a video camera (JVC Everio, GZ-MG130E, Victor Company of Japan Limited, Yokohama, Japan). The "VirtualDub" software (freely available at http://www.virtualdub.org) allowed for a one-by-one picture analysis of the video recordings in order to locate the ball contact in the Perspex target or the soft floor mat and the wooden plank. The video image showing the impact of the ball was subsequently analyzed digitally to determine the distance from the target to the nearest centimeter.

### Statistical analysis

Descriptive statistics included information on averages, medians, standard deviations and 90% confidence intervals (CI 90%). Statistical analyses were performed using the IBM SPSS Statistics version 22 (IBM Corporation, New York, USA) and included testing for normal distribution and homoscedasticity by means of the Shapiro-Wilk and Levene’s test, respectively. As the requirements of normal distribution and homogeneity of variance were met, statistical analyses were performed using the t-test for paired samples. The alpha-level for significance was set at *p*<0.05. Based on an *α*-error probability of 0.05, an estimated effect size of *d*=0.5 and a 1−*β* error probability of 0.8 a total sample size of n=26 would be required to detect a statistically significant difference.

Reliability of velocity and accuracy parameters with and without KT were estimated by calculating intra-class correlation coefficients (ICC; Model C, two-way fixed, computing consistency) (Muller and Buttner, 1994). Therefore, the data set of each participant was split into two means by the following procedure: three single shots/throws were randomly chosen and averaged for the first mean, additional but different three shots/throws were randomly chosen and averaged for the second mean.

For soccer and handball, the attempt with the lowest ball speed and the corresponding value for accuracy performance was excluded from data analysis, if the sum of this shot and the standard deviation of all trials in the taped or the untaped condition was less than the attempt with the second lowest ball speed, in order to eliminate confounders, e.g. a slight but not obvious slip or a tumble before the goal shot. This resulted in 161 and 164 data sets in soccer, as well as in 183 and 183 data sets in handball for the KT and no tape condition, respectively. For each player, a mean value was calculated for velocity and accuracy in the taped and untaped condition and analyzed for evaluation of KT application effects. For distinguishing players with low or high ball speeds in shooting and throwing, the median value for the attempts in the untaped condition was calculated. Depending on these results, players were either assigned to the low or high performance group.

Furthermore, effect statistics were calculated in order to differentiate between a beneficial, trivial or harmful effect between the taped und untaped condition according to Batterham and Hopkins (2006). The threshold value representing benefit and harm was determined by calculating the minimum clinically important difference (MCID) by means of a distribution-based approach using Cohen’s effect size *d* (Copay et al., 2007). To discriminate between a beneficial and harmful effect, a threshold value of 20% of the pooled standard deviation of attempts with and without KT was chosen, corresponding to a small effect size (Cohen’s *d*). The pooled standard deviation of both groups was calculated by the formula:

$$SD=\sqrt{\frac{(N_{KT}-1)*{SD}_{KT}2+(N_{NT}-1)*{SD}_{NT}2}{N_{KT}+N_{NT}-2}}$$

The probability that an effect of KT application was a decrease, trivial or an increase of the observed parameter was expressed as: <0.5% = most unlikely or almost certainly not; 0.5–5% = very unlikely; 5–25% = unlikely or probably not; 25–75% = possibly; 75–95% = likely or probably; 95–99.5% = very likely; >99.5% = most likely or almost certainly (Hopkins, 2007; Hopkins et al., 2009). Values above 5% for both beneficial and harmful effects of tape applications indicated that this effect was mechanistically unclear.

## Results

Shooting and throwing with maximal velocity demonstrated good to excellent reliability in soccer (ICC=0.89 with KT and ICC=0.93 without KT) and handball (ICC=0.98 with KT and ICC=0.97 without KT). Shooting and throwing accuracy revealed generally poor reliability in soccer (ICC=0.36 with KT and ICC=−0–27 without KT) and handball (ICC=0.59 with KT and ICC=−0.34 without KT). The majority of participants felt the tapes to be pleasant or at least neutral. The players’ perceptions of the KT application on the skin are presented in Table 1.

### Effects of KT applications on shooting and throwing performance

In amateur soccer players, KT applications induced a significant increase in ball speed of 1.4 ±3.5 km/h with 16 of the 26 players (=62%) achieving higher average velocities (Table 2). Furthermore, accuracy performance was significantly improved by KT applications as indicated by an average decrease of 6.9 ±16.1 cm distance from the target. The probability of a beneficial effect of kinesiotape applications was 74% for ball speed and 56% for accuracy performance.

KT applications in amateur handball players significantly increased ball speed by 1.2 ±2.5 km/h with 23 of the 32 handball players (=72%) achieving higher values. The likelihood of a positive effect of kinesiotape applications was 88% (likely beneficial), while accuracy performance simultaneously decreased with a significantly greater distance from the target of 3.4 ±6.4 cm, indicating a very likely negative effect (95%).

### Effects of KT applications with respect to the performance level

Median ball speed without KT was 78.3 km/h in soccer and 74.8 km/h in handball players. These values were used as a distinguishing criterion for the allocation to the groups with high and low performance athletes. Low performing athletes demonstrated significant gains in ball velocities after KT applications compared with non-significant gains in high performing athletes (Table 3). The opposite was found for shooting and throwing accuracy: KT applications in high performing athletes induced no meaningful effects, while KT in low performing athletes induced a significant deterioration with regard to the accuracy task in handball.

## Discussion

The purpose of this study was to evaluate the effects of kinesiotape (KT) applications on shooting and throwing performance in amateur soccer and handball players, respectively, compared to a no-tape condition. Ball velocities were considerably lower in comparison with previously published data of professional athletes for soccer kicks with 29 m/s or 104 km/h (van den Tillaar and Ulvik, 2014; Andersen and Dorge, 2011) and handball throws with 25 m/s or 90 km/h (Gorostiaga et al., 2005; Rojas et al., 2012), indicating the amateur level of the players enrolled in this study. With a probability of 74% in soccer and 88% in handball, KT applications are likely to improve ball speed when shooting or throwing at maximum effort. This could prove to be an advantage in team sports, where it is important to achieve maximum ball velocity. However, a potential loss in accuracy could counterbalance an increase of ball velocity.

Currently, research lacks scientific support for beneficial effects of KT with regard to muscle facilitation in the upper and lower extremity in healthy subjects (Fu et al., 2008; Chang et al., 2010), being a relevant indicator for shooting and throwing velocity in soccer and handball. Of note, a recent study found that KT applied to the vastus medialis did not alter peak torque generation, but shortened the time to generate peak torque in healthy participants (Wong et al., 2012). This effect can be considered to be crucial for kicking and throwing ball velocity and is in line with the gains in ball speed we observed in soccer kicks and handball throws.

On the other hand, the gain in ball speed was accompanied by a questionable effect on accuracy in soccer, but a significant increase in ball speed in handball was counteracted by a very likely negative effect (95%) on accuracy performance, outweighing the benefits of increased throwing ball velocities. Due to the importance of accuracy especially in shots/throws from a larger distance, the loss of accuracy is more relevant when shots/throws from a larger distance have to be taken. Thus, a decision to use KT could be different e. g. for pivots or back players. In this context, different roles of proximal joints like the shoulder, determining the trajectory of the hand in space, and distal joints like the fingers releasing the ball have to be considered (Hore et al., 1996). We therefore hypothesized that if concentric muscle actions of the shoulder were increased by means of KT applications, these probably induced a significant perturbation of the movement routine with fingers opening too soon or too late, thus altering the moment of ball release with a negative impact on accuracy performance. Based on our results, this deterioration of coordinated proximal-to-distal sequencing is supposed to be more evident in handball, where shoulder joint kinematics are affected to a greater extent by surrounding muscles compared to the hip and knee joints in soccer, and the wrist and fingers adding a further variable to the accuracy task as opposed to the foot in soccer. This would explain differences found for accuracy performance between soccer and handball players after KT application. Potentially, practicing with applied KT and therefore increased throwing velocity could reduce the loss of accuracy over time. This potential effect of familiarization to KT should be addressed in future studies.

The present results indicate that especially players with lower kicking and throwing skills may benefit from KT applications in terms of ball velocity gains. To our knowledge, there is no study that had previously picked up this idea of investigating the effect of KT in different levels of performance. As the mode of action of muscular KT applications is primarily of proprioceptive nature (Murray and Husk, 2001), we assumed that the effects, most of which were attained for ball speed, may be explained with an activation of cutaneous sensors and consecutive strengthening of afferences from the periphery, increasing the recruitment of motor units which consecutively increased muscle tone. We further hypothesized that players with higher kicking and throwing ball velocities already demonstrated a higher percentage of motor unit recruitment compared with low performing athletes. Hence, the potential of lower skilled players to increase kicking or throwing ball speed by means of a cutaneous stimulation from KT applications was likely to be higher.

In this context, the application of KT in healthy amateur and professional players aiming at athletic performance enhancement has to be questioned. These athletes presumably present reasonable muscle strength levels and athleticism, where KT has little benefit. Hence, we rather assumed that KT might be a promising supportive approach in injury rehabilitation or musculoskeletal disorders, where muscle weakness and strength imbalances were common conditions. However, as we included only healthy participants, the potential benefits of KT in prevention and rehabilitation has to be addressed in future studies.

Lastly, the effectiveness of a technical support like KT depends on its perception to a large extent, i.e. comfort of the tapes. A supportive technique that is uncomfortable will not be accepted by an athlete in the long run and is therefore unlikely to be implemented in the athlete’s routine, independent of a potential performance improvement. Hence, it is essential to note that only two participants in this study reported an adverse perception of KT after its application. However, subgroups were too small for further statistical analyses to evaluate the effect of KT perception on kicking or throwing performance.

## Limitations

ICCs prove high reliability for velocity measurement in soccer and handball. In contrast, reliability for accuracy measurements is weak, reflecting the common problems with assessments of accuracy in ball games, when the aim is to shoot/throw fast and precise (Kellis and Katis, 2007). Interestingly, reliability improved in taped conditions compared to conditions without tape. Possibly, this could indicate that the tape improves accuracy in accuracy tasks. However, the results in accuracy should be considered with caution taking the overall weak reliability into account. Furthermore, blinding of participants and observers was impossible. It has to be taken into account that athletes at all levels are susceptible to the placebo effect. This effect may improve shooting and throwing performance in soccer and handball players, but it even may disguise deteriorated effects on performance, just because participants are aware of being taped. For this reason, several trials were performed to minimize the impact of a potential placebo effect due to KT applications on performance. In addition, KT application of the m. subscapularis is difficult due to its anatomical site. However, the tape was attached simulating an internal rotation of the shoulder joint, being characteristic for throwing tasks and corresponding to the main function of the m. subscapularis. Certainly, it is possible that different KT attachments could have a larger impact on performance in shooting (e.g. hip extensors, knee flexors of the kicking leg, or muscular tapes of the stance leg, pelvis or trunk) and throwing (e.g. the deltoid muscle, drive leg, pelvis or trunk).

## Practical Applications

The results of the present investigation demonstrate that KT applications may improve ball speed in male amateur athletes in soccer and handball. While the beneficial effect of KT on ball speed was not counteracted by a deteriorated accuracy performance in kicking with maximum effort and therefore appears worthwhile, the gain in handball throwing velocity is likely at the expense of accuracy. Thus, amateur soccer players might benefit from KT. Due to the conflicting results in the throwing performance, handball players have to balance the gain in velocity and the loss in accuracy when deciding to use KT.

## Figures and Tables

**Table 1:** Perception of KT application on the skin

	Soccer (n=26)	Handball (n=31)*
very pleasant	4	2
pleasant	12	14
neutral	9	14
unpleasant	1	1
very unpleasant	0	0

* One player did not respond (handball)

**Table 2:** Performance changes induced by KT applications by comparing untaped and taped conditions

	Mean „no tape“ (SD)	Mean „KT“(SD)	Mean change (90% CI)	Chances (%) that the effect of KT is beneficial/trivial/negative	*p*
Soccer (n=26)					
Speed (km/h)	78.0 (6.9)	79.4 (7.6)	+1.4 (0.3 to 2.6)	74/26/0 possibly beneficial	0.047
Distance from the target (cm)	64.0 ( 11.0)	57.1 (9.4)	−6.9 (−12.3 to −1.5)	56/45/0 possibly beneficial	0.039

Handball (n=32)					
Speed (km/h)	75.9 (7.7)	77.0 (6.9)	+1.2 (0.4 to 1.9)	88/12/0 likely beneficial	0.013
Distance from the target (cm)	28.0 (5.4)	31.5 (7.6)	+3.4 (1.5 to 5.4)	0/5/95 very likely negative	0.005

**Table 3:** Effects of KT application on ball speed and accuracy depending on performance levels

	Mean change (SD)	Mean change 90% CI	Chances (%) that the effect of KT is beneficial/trivial/negative	*p*
Soccer n=26				
Ball speed (km/h)				
*Low performing (<78.3 km/h) n=13*	+1.7 (2.4)	0.5 to 2.9	93/7/0 likely beneficial	0.028
*High performing (>78.3 km/h) n=13*	+1.2 (4.5)	−1.0 to +3.4)	49/48/4 possibly beneficial	0.346
Distance from target (cm)				
*Low performing (<78.3 km/h) n=13*	−5.4 (17.5)	−14.1 to 3.2	18/81/0 likely trivial	0.284
*High performing (>78.3 km/h) n=13*	−8.3 (15.2)	−15.8 to −0.8	48/52/0 possibly trivial	0.072

Handball n=32				
Ball speed (km/h)				
*Low performing (<74.8 km/h) n=16*	+1.8 (1.7)	1.1 to 2.6	100/0/0 almost certainly beneficial	0.001
*High performing (>74.8 km/h) n=16*	+0.5 (3.0)	−0.8 to +1.8	34/61/5 possibly trivial	0.511
Distance from target (cm)				
*Low performing (<74.8 km/h) n=16*	+4.1 (5.7)	1.7 to 6.6	0/5/95 likely negative	0.011
*High performing (>74.8 km/h) n=16*	+2.7 (7.1)	−0.4 to 5.9	1/45/55 possibly negative	0.147

## Abbreviations

CI: confidence interval
ICC: intra-class correlation
KT: kinesiotape
m.: musculus
MCID: minimum clinically important difference
N: number of subjects
NT: no tape
SD: standard deviation

## References

[b1-jhk-49-119] Andersen TB, Dorge HC (2011). The influence of speed of approach and accuracy constraint on the maximal speed of the ball in soccer kicking. Scand J Med Sci Sports.

[b2-jhk-49-119] Batterham AM, Hopkins WG (2006). Making meaningful inferences about magnitudes. Int J Sports Physiol Perform.

[b3-jhk-49-119] Briem K, Eythorsdottir H, Magnusdottir RG, Palmarsson R, Runarsdottir T, Sveinsson T (2011). Effects of kinesio tape compared with nonelastic sports tape and the untaped ankle during a sudden inversion perturbation in male athletes. J Orthop Sports Phys Ther.

[b4-jhk-49-119] Chang HY, Chou KY, Lin JJ, Lin CF, Wang CH (2010). Immediate effect of forearm Kinesio taping on maximal grip strength and force sense in healthy collegiate athletes. Phys Ther Sport.

[b5-jhk-49-119] Copay AG, Subach BR, Glassman SD, Polly DW, Schuler TC (2007). Understanding the minimum clinically important difference: a review of concepts and methods. Spine J.

[b6-jhk-49-119] Csapo R, Alegre LM (2015). Effects of Kinesio taping on skeletal muscle strength-A meta-analysis of current evidence. J Sci Med Sport.

[b7-jhk-49-119] Csapo R, Herceg M, Alegre LM, Crevenna R, Pieber K (2012). Do kinaesthetic tapes affect plantarflexor muscle performance?. J Sports Sci.

[b8-jhk-49-119] Dorge HC, Andersen TB, Sorensen H, Simonsen EB, Aagaard H, Dyhre-Poulsen P, Klausen K (1999). EMG activity of the iliopsoas muscle and leg kinetics during the soccer place kick. Scand J Med Sci Sports.

[b9-jhk-49-119] Drouin JL, McAlpine CT, Primak KA, Kissel J (2013). The effects of kinesiotape on athletic-based performance outcomes in healthy, active individuals: a literature synthesis. J Can Chiropr Assoc.

[b10-jhk-49-119] Firth BL, Dingley P, Davies ER, Lewis JS, Alexander CM (2010). The effect of kinesiotape on function, pain, and motoneuronal excitability in healthy people and people with Achilles tendinopathy. Clin J Sport Med.

[b11-jhk-49-119] Fu TC, Wong AM, Pei YC, Wu KP, Chou SW, Lin YC (2008). Effect of Kinesio taping on muscle strength in athletes-a pilot study. J Sci Med Sport.

[b12-jhk-49-119] Gorostiaga EM, Granados C, Ibanez J, Izquierdo M (2005). Differences in physical fitness and throwing velocity among elite and amateur male handball players. Int J Sports Med.

[b13-jhk-49-119] Gusella A, Bettuolo M, Contiero F, Volpe G (2014). Kinesiologic taping and muscular activity: a myofascial hypothesis and a randomised, blinded trial on healthy individuals. J Bodyw Mov Ther.

[b14-jhk-49-119] Hopkins WG (2007). A spreadsheet for deriving a confidence interval, mechanistic inference and clinical inference from a P value. Sportscience.

[b15-jhk-49-119] Hopkins WG, Marshall SW, Batterham AM, Hanin J (2009). Progressive statistics for studies in sports medicine and exercise science. Med Sci Sports Exerc.

[b16-jhk-49-119] Hore J, Watts S, Tweed D, Miller B (1996). Overarm throws with the nondominant arm: kinematics of accuracy. J Neurophysiol.

[b17-jhk-49-119] Hsu YH, Chen WY, Lin HC, Wang WT, Shih YF (2009). The effects of taping on scapular kinematics and muscle performance in baseball players with shoulder impingement syndrome. J Electromyogr Kinesiol.

[b18-jhk-49-119] Huang CY, Hsieh TH, Lu SC, Su FC (2011). Effect of the Kinesio tape to muscle activity and vertical jump performance in healthy inactive people. Biomed Eng Online.

[b19-jhk-49-119] Kellis E, Katis A (2007). Biomechanical characteristics and determinants of instep soccer kick. J Sports Sci Med.

[b20-jhk-49-119] Lins CA, Neto FL, Amorim AB, Macedo Lde B, Brasileiro JS (2013). Kinesio Taping(®) does not alter neuromuscular performance of femoral quadriceps or lower limb function in healthy subjects: randomized, blind, controlled, clinical trial. Man Ther.

[b21-jhk-49-119] Lyle MA, Sigward SM, Tsai LC, Pollard CD, Powers CM (2011). Influence of maturation on instep kick biomechanics in female soccer athletes. Med Sci Sports Exerc.

[b22-jhk-49-119] Merino-Marban R, Mayorga-Vega D, Fernandez-Rodriguez E (2013). Effect of kinesio tape application on calf pain and ankle range of motion in duathletes. J Hum Kinet.

[b23-jhk-49-119] Muller R, Buttner P (1994). A critical discussion of intraclass correlation coefficients. Stat Med.

[b24-jhk-49-119] Murray H, Husk LJ (2001). Effect of kinesio™ taping on proprioception in the ankle. J Orthop Sports Phys Ther.

[b25-jhk-49-119] Nakajima MA, Baldridge C (2013). The effect of kinesio® tape on vertical jump and dynamic postural control. Int J Sports Phys Ther.

[b26-jhk-49-119] Raeder C, Fernandez-Fernandez J, Ferrauti A (2015). Effects of Six Weeks of Medicine Ball Training on Throwing Velocity, Throwing Precision, and Isokinetic Strength of Shoulder Rotators in Female Handball Players. J Strength Cond Res.

[b27-jhk-49-119] Rojas FJ, Gutierrez-Davila M, Ortega M, Campos J, Parraga J (2012). Biomechanical analysis of anticipation of elite and inexperienced goalkeepers to distance shots in handball. J Hum Kinet.

[b28-jhk-49-119] Rousanoglou E, Noutsos K, Bayios I, Boudolos K (2014). Ground reaction forces and throwing performance in elite and novice players in two types of handball shot. J Hum Kinet.

[b29-jhk-49-119] Schiffer T, Mollinger A, Sperlich B, Memmert D (2015). Kinesio taping and jump performance in elite female track and field athletes and jump performance in elite female track and field athletes. J Sport Rehabil.

[b30-jhk-49-119] Slupik A, Dwornik M, Bialoszewski D, Zych E (2007). Effect of Kinesio Taping on bioelectrical activity of vastus medialis muscle. Preliminary report. Ortop Traumatol Rehabil.

[b31-jhk-49-119] Thelen MD, Dauber JA, Stoneman PD (2008). The clinical efficacy of kinesio tape for shoulder pain: a randomized, double-blinded, clinical trial. J Orthop Sports Phys Ther.

[b32-jhk-49-119] Tsai HJ, Hung HC, Yang JL, Huang CS, Tsauo JY (2009). Could Kinesio tape replace the bandage in decongestive lymphatic therapy for breast-cancer-related lymphedema? A pilot study. Support Care Cancer.

[b33-jhk-49-119] van den Tillaar R, Ulvik A (2014). Influence of instruction on velocity and accuracy in soccer kicking of experienced soccer players. J Mot Behav.

[b34-jhk-49-119] Vercelli S, Sartorio F, Foti C, Colletto L, Virton D, Ronconi G, Ferriero G (2012). Immediate effects of kinesiotaping on quadriceps muscle strength: a single-blind, placebo-controlled crossover trial. Clin J Sport Med.

[b35-jhk-49-119] Vered E, Oved L, Zilberg D, Kalichman L (2015). Influence of kinesio tape application direction on peak force of biceps brachii muscle: A repeated measurement study. Journal of Bodywork & Movement Therapies.

[b36-jhk-49-119] Wagner H, Pfusterschmied J, Von Duvillard SP, Muller E (2012). Skill-dependent proximal-to-distal sequence in team-handball throwing. J Sports Sci.

[b37-jhk-49-119] Wong OM, Cheung RT, Li RC (2012). Isokinetic knee function in healthy subjects with and without Kinesio taping. Phys Ther Sport.

[b38-jhk-49-119] Yasukawa A, Patel P, Sisung C (2006). Pilot study: investigating the effects of Kinesio Taping in an acute pediatric rehabilitation setting. Am J Occup Ther.

[b39-jhk-49-119] Young WB, Rath DA (2011). Enhancing foot velocity in football kicking: the role of strength training. J Strength Cond Res.

